# Association between the lactate-to-albumin ratio and 28-day all-cause mortality in diabetic ketoacidosis patients: A retrospective cohort study utilizing the MIMIC-IV database

**DOI:** 10.1371/journal.pone.0344767

**Published:** 2026-03-12

**Authors:** Fan Zhang, Lingchen Wei, Yuan Liu, Guang Yang, Xiaobin Zhao, Runyun Zhang

**Affiliations:** 1 Guang’anmen Hospital, China Academy of Chinese Medical Sciences, Beijing, China; 2 College of Traditional Chinese Medicine, Shandong University of Traditional Chinese Medicine, Jinan, China; 3 XuanWu TCM Hospital Beijing, Beijing, China; 4 Beijing Jingmei Group General Hospital, Beijing, China; University of Connecticut, UNITED STATES OF AMERICA

## Abstract

**Objective:**

The objective of this study was to assess the relationship between the lactate-to-albumin ratio upon hospital admission and the clinical outcomes of critically ill patients with a diagnosis of diabetic ketoacidosis.

**Methods:**

A retrospective cohort analysis was conducted. Patients were classified into two groups according to their lactate-to-albumin ratio values: low-lactate-to-albumin ratio (< 0.75) and high-lactate-to-albumin ratio (≥ 0.75). The association between lactate-to-albumin ratio and mortality was evaluated using Cox proportional hazards regression models and restricted cubic spline curves. Receiver operating characteristic curves were employed to assess the diagnostic capability of lactate-to-albumin ratio in predicting prognosis. Additionally, Kaplan-Meier survival analysis was performed to compare the cumulative survival rates between two groups. Subgroup analyses were conducted to validate the robustness of the findings.

**Conclusion:**

A high lactate-to-albumin ratio (≥ 0.75) at admission was recognized as an independent risk factor for 28-day all-cause mortality in diabetic ketoacidosis patients.

## 1 Introduction

Diabetic ketoacidosis (DKA) represents a prevalent and serious acute metabolic complication in individuals with diabetes, serving as a leading cause of hospital admissions in this patient population. Between 2009 and 2014, the estimated average annual hospitalization rate for DKA increased by 54.9 percent (from 19.5 to 30.2 per 1,000), according to a U.S. epidemiological study [1]. Despite advancements in medical technology and the widespread use of insulin, which have reduced the overall mortality rate of DKA to less than 1%, patients aged over 60 years and those with complex comorbidities or additional complications continue to exhibit higher mortality and readmission rates [2]. Statistical data indicate that the average hospitalization cost for an individual diagnosed with DKA in the United States in 2017 was approximately $30,836, causing a considerable economic strain on both individual patients as well as healthcare systems [3]. Consequently, identifying reliable prognostic markers is critically important for the early recognition of high-risk DKA patients, enhancing clinical outcomes, and alleviating the associated healthcare burden. Recent studies have explored the prognostic utility of composite biomarkers such as the creatinine-to-albumin ratio (CAR) in sepsis-associated acute kidney injury [4] and the neutrophil-to-prognostic nutritional index ratio (NPNR) in elderly sepsis patients [5], underscoring the importance of metabolic-inflammatory interactions in risk stratification. In addition, the lactate-to-albumin ratio (LAR) has also proved itself to be a hopeful prognostic indicator, garnering increasing attention in clinical research. Anaerobic metabolism produces lactate, which is mainly metabolized in the liver, reflecting tissue hypoxia and hypoperfusion [6]. Albumin is an indicator of the degree of inflammation and infection [7], but both levels are susceptible to other factors, which may reduce their accuracy as prognostic markers. LAR combines the biological characteristics of both to more accurately reflect the body’s metabolic level. In 2015, Wang et al. were the first to introduced the LAR，and they hold the view that the LAR has the possibility to act as a predictor for mortality rate and organ malfunction, demonstrating that mature individuals afflicted with advanced sepsis and infectious shock, who exhibited elevated LAR levels were at a significantly higher risk of developing multiple organ dysfunction syndrome (MODS) and experiencing increased mortality [8]. The LAR has been demonstrated to serves as an separate prognostic marker for conditions such as acute pancreatitis, acute myocardial infarction, and other related diseases [9,10]. However, there are still relatively few researches on the role of LAR plays in DKA patients and its correlation with all-cause mortality. Given the present state of research, the objective of our study was to assess the role of LAR in predicting all-cause mortality among critically ill patients with DKA through an analysis of the Critical Care Medicine Information Mart-IV (MIMIC-IV) database.

## 2 Materials and methods

### 2.1 Data source and study population

This research adopts a backward-looking cohort design, all data were obtained from the MIMIC database. All personal information in this database is deidentified and therefore does not require informed consent or ethical approval from patients. The second author of this study (Lingchen Wei) completed the Cooperative Institutional Training Program (CITI) course and passed the “Conflict of Interest” and “Data or sample Only Research” exams (ID: 61829958) to be eligible to extract data from this database.

The MIMIC-IV database comprises a single-center dataset derived from Beth Israel Deaconess Medical Center, encompassing comprehensive statistics about patients received in the intensive care unit (ICU) over the years 2008–2022. The most recent version, designated as v3.1, was officially released in October 2024. The developers of the database show that MIMIC-IV was created following best practices in scientific computing. Validation of the build process assessed data integrity, data consistency, and de-identification [11]. The MIMIC database has been extensively validated and has been adopted by several peer-reviewed high-impact medical journal articles, confirming its reliability and validity in clinical studies.

**Fig 1:**
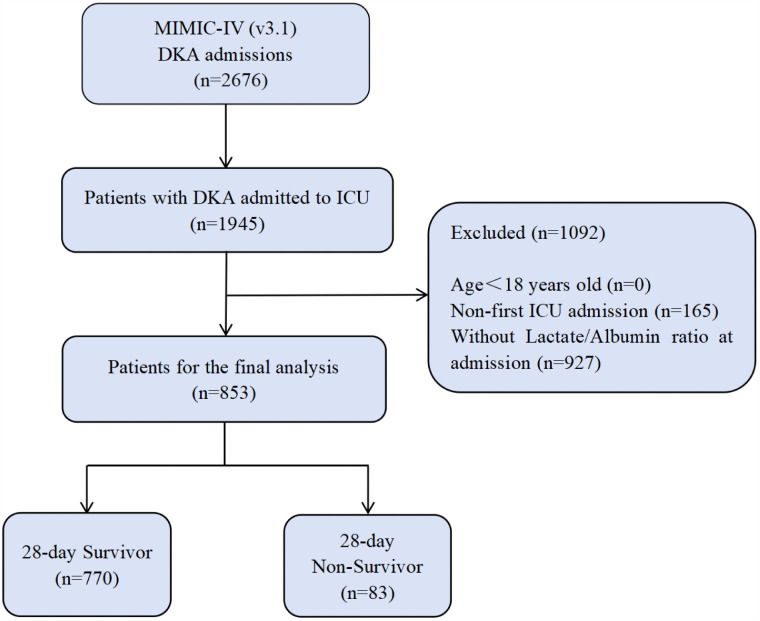
Flowchart of inclusion and exclusion.

Admission information of DKA patients was extracted based on the codes of the 9th and 10th incarnations of the International Classification of Diseases. A total of 2,676 patients in the MIMIC database were enrolled, among whom 1,945 had been hospitalized in the ICU. Upon subsequent filtering, patients meeting any of the criteria as follows were excluded from the analysis (Fig 1): (1) Individuals under 18 years old on their initial hospital admission; (2) Patients whose ICU admission was not their first; (3) Patients with several admissions to the ICU, for whom the analysis was only conducted on the data of their first admission; (4) Patients without lactate and albumin values measured within the initial 24 hours after being admitted. A final cohort of 853 patients was incorporated into this study.

### 2.2 Data extraction process

We applied Structured Query Language (SQL) to sift out variables during the initial 24 hours after being admitted from the MIMIC-IV database. The LAR served as the primary study variable, defined as the quotient of lactate divided by albumin. The extracted data of blood lactate and blood albumin were all the first test results during the initial 24 hours after being admitted to reduce the interference of subsequent treatment on the values of lactate and albumin. The extracted potential confounding factor variables include six major categories: (1) Demographic information: age, gender, and race; (2) Vital signs: heart rate (HR), systolic blood pressure (SBP), diastolic blood pressure (DBP), respiratory rate, and body temperature; (3) Laboratory indicators: Hematocrit, white blood cell (WBC), hemoglobin, bicarbonate, platelets, serum urea nitrogen, serum creatinine, blood glucose, serum sodium, serum potassium, serum chloride, lymphocyte percentage, prothrombin time, international normalized ratio, serum lactate, and serum albumin. All laboratory test results were recorded in the initial assessment upon patient admission, namely within the period ranging from 6 hours prior to admission to the initial 24 hours after admission. (4) Comorbidities: congestive heart failure, atrial fibrillation, respiratory failure, peripheral vascular disease, hypertension, renal disease, sepsis; (5) Clinical treatments: Vitamin B1, sodium bicarbonate, potassium chloride, vasopressin, mechanical ventilation; (6) Disease severity scores: acute physiology score III (APS III), sequential organ failure assessment (SOFA).

### 2.3 Treatment of missing values

To avoid bias, when the proportion of missing values for a variable was less than 5% (such as body temperature, serum glucose, hematocrit, white blood cells, hemoglobin, and platelets), the missing values were substituted with the average value. For variables having missing values that exceed 5% and are below 15% (such as the percentage of lymphocytes), there was an implementation of multiple imputation by employing the mice package within the R statistical software environment; Variables with missing values exceeding 15% (prothrombin time, international normalized ratio) were excluded from the analysis.

### 2.4 Grouping and clinical outcomes

In this study, patients were stratified into two groups: the group surviving for 28 days (n = 770) and the group with mortality within 28 days (n = 83). The primary outcome of the study was 28-day all-cause mortality following hospital admission. Furthermore, using the optimal cutoff value derived from the receiver operating characteristic (ROC) curve, patients were categorized into a low LAR group (< 0.75) and a high LAR group (≥ 0.75).

### 2.5 Statistical analyses

When analyzing the baseline characteristics, the distribution of continuous variables was evaluated by using the Kolmogorov-Smirnov test. We analyzed continuous variables with the normal distribution by using either the Student’s t-test or one-way analysis of variance (ANOVA), the outcomes are reported as mean ± standard deviation. Continuous variables which did not conform to a normal distribution were analyzed by using the Mann-Whitney U test or the Kruskal-Wallis test and were presented as the median (interquartile range, IQR). Categorical variables were analyzed for comparison by using the Pearson chi-square (χ2) test and expressed as proportions (%). Potential risk factors were initially identified through univariate Cox regression analysis, and variables with P values ＜0.05 were subsequently incorporated into multivariate Cox regression models to recognize independent predictors of in-hospital mortality. Simultaneously, Additionally, Cox proportional hazards model was utilized to calculate the hazard ratio (HR) and its corresponding 95% confidence interval (CI) for the connection between the LAR and the study endpoint. Moreover, in accordance with the univariate Cox analysis, confounding variables with P values < 0.05 were adjusted. Model I: Unadjusted; Model II: Adjusted for age, race, SBP, DBP, respiratory rate, CHF, respiratory failure, AF, hypertension, and sepsis; Model III: Adjusted for age, race, SBP, DBP, respiratory rate, CHF, AF, respiratory failure, hypertension, sepsis, hematocrit, hemoglobin, platelets, serum urea nitrogen, serum creatinine, serum sodium, serum potassium, serum chloride, bicarbonate, vasopressin, SOFA, and APSIII. The proportional hazards assumption was tested using the Schoenfeld residuals method. The results showed that no significant violation of this assumption was found, indicating that the model is highly applicable. We utilized ROC curve analysis to evaluate the prognostic performance of lactate, albumin and LAR for prognosis, and determined the cut-off value of LAR in accordance with the Youden index, which was subsequently utilized to categorize patients into high-LAR and low-LAR groups. Then, unadjusted survival curves were generated using the Kaplan-Meier (K-M) method, and the log-rank test was applied to analyze the differences between the two curves. The restricted cubic spline regression model (RCS) was applied to reveal the non-linear association between the hospital mortality risk rate and LAR. Finally, subgroup analyses were performed to examine potential variations in the effects of the LAR across different patient subgroups, including gender, race, sodium bicarbonate, vasopressin, congestive heart failure, respiratory failure, atrial fibrillation, hypertension, and sepsis. Our findings were visualized using a forest plot. All analyses were performed using SPSS version 27.0 and R package version 4.4.2, with statistical significance defined as a two-tailed P-value < 0.05.

## 3 Result

### 3.1 Comparison of baseline characteristics between two groups segmented by 28-day mortality (Table 1)

In total, there were 853 patients satisfied the inclusion standards and were classified based on 28-day mortality. The 853 patients were stratified into the survival group (770, 90.27%) and the non-survival group (83, 9.73%), consisting of 418 (49%) females and 435 (51%) males. The age at the median was 54.0 (38.0, 66.0) years. A total of 487 (57%) white patients were hospitalized. The 28-day mortality rate after admission was 9.73%. We observed that patients in the group of non-survival were remarkably more aged than patients in the survival group (P < 0.001); the respiratory rate, SOFA and APSIII were higher, while SBP and DBP were lower (P < 0.001). Patients with combined respiratory failure, hypertension and sepsis were more common, accounting for 66.27% (P < 0.001), 78.31% (P = 0.004) and 50.60% (P < 0.001) of the non-survival group respectively, but those with combined congestive heart failure and atrial fibrillation only accounted for 37.35% and 22.89% of the non-survival group (P < 0.001). In the group of non-survival, the proportion of patients receiving vitamin B1, sodium bicarbonate and vasopressin treatment was markedly lower, accounting for 0% (P = 0.012), 38.55% (P = 0.005) and 42.17% (P < 0.001) of the non-survival group patients respectively. Laboratory findings revealed that, upon admission, the LAR non-survival group (1.20 0.80, 2.10) was markedly higher than survival group (0.50 0.40, 0.90) (P < 0.01), and serum glucose, serum creatinine, BUN, Sodium, Potassium, Chloride and lactate were markedly higher than patients in the survival group (P < 0.005). Compared to the survival group, the non-survival group exhibited markedly lower levels of hematocrit, platelet count, hemoglobin, and albumin (P < 0.005), while no remarkable differences were found in the remaining covariates for two groups (P > 0.05).

**Table 1:** Comparison of baseline characteristics between two groups segmented by 28-day mortality.

Variables	All patients (n = 853)	Survival group (n = 770)	Non-survival group (n = 83)	Statistic t/Z/χ2	P- Value
**Characteristics**					
Age, year	54.00 (38.00, 66.00)	52.00 (38.00, 64.00)	67.00 (57.00, 74.00)	Z = −6.91	**<.001**
Gender, n(%)				χ² = 1.17	**0.280**
Female	418 (49.00)	382 (49.61)	36 (43.37)		
Male	435 (51.00)	388 (50.39)	47 (56.63)		
Race, n(%)				χ² = 16.50	**<.001**
White	487 (57.09)	444 (57.66)	43 (51.81)		
Black	235 (27.55)	220 (28.57)	15 (18.07)		
Other	131 (15.36)	106 (13.77)	25 (30.12)		
**Vital sign**					
Heart Rate,beats/min	93.03 ± 15.30	92.82 ± 14.56	95.00 ± 20.97	t = −0.92	**0.359**
SBP,mmHg	120.60 (109.90, 133.60)	121.60 (110.73, 134.38)	109.70 (103.75, 120.85)	Z = −5.49	**<.001**
DBP,mmHg	66.80 (59.20, 75.80)	67.40 (59.80, 76.47)	61.60 (54.95, 68.10)	Z = −5.05	**<.001**
Respiratory Rate,beats/min	19.20 (16.90, 22.00)	19.00 (16.80, 21.60)	22.40 (18.55, 27.05)	Z = −5.41	**<.001**
Temperature,°C	36.90 (36.70, 37.10)	36.90 (36.70, 37.10)	36.80 (36.40, 37.40)	Z = −1.57	0.116
**Laboratory parameters**					
Hematocrit	31.86 ± 6.60	32.04 ± 6.44	30.20 ± 7.77	t = 2.08	**0.041**
WBC,K/uL	10.30 (7.30, 14.00)	10.10 (7.30, 13.78)	12.30 (6.95, 17.30)	Z = −1.90	**0.058**
Hemoglobin, g/dL	10.40 (8.90, 12.10)	10.50 (9.00, 12.10)	9.50 (7.70, 11.20)	Z = −3.47	**<.001**
Platelets, K/uL	211.00 (154.00, 268.00)	216.00 (160.00, 271.00)	167.00 (110.00, 230.50)	Z = −4.31	**<.001**
Bicarbonate,mmol/L	12.00 (8.00, 17.00)	12.00 (7.00, 17.00)	13.00 (8.50, 17.00)	Z = −1.31	**0.189**
BUN, mg/dL	19.00 (10.00, 35.00)	17.00 (10.00, 33.00)	38.00 (22.50, 59.00)	Z = −6.81	**<.001**
Scr,mg/dL	1.10 (0.70, 1.80)	1.00 (0.70, 1.60)	1.90 (1.20, 2.90)	Z = −6.20	**<.001**
Glucose,mg/dL	209.70 (181.20, 245.60)	207.55 (180.17, 242.28)	234.20 (198.40, 287.00)	Z = −3.83	**<.001**
Sodium, mEq/L	133.00 (129.00, 136.00)	132.00 (129.00, 136.00)	136.00 (130.50, 139.00)	Z = −4.05	**<.001**
Potassium, Eq/L	3.70 (3.30, 4.00)	3.70 (3.30, 4.00)	3.80 (3.35, 4.10)	Z = −2.10	**0.036**
Chloride, mmol/L	96.00 (89.00, 101.00)	96.00 (89.00, 101.00)	98.00 (92.50, 104.50)	Z = −3.21	**0.001**
Lymphocytes	1.10 (0.80, 1.70)	1.10 (0.80, 1.70)	1.10 (0.65, 1.70)	Z = −0.79	0.429
Lactate,mmol/L	2.00 (1.40, 3.30)	1.90 (1.30, 3.10)	3.50 (2.40, 5.10)	Z = −7.58	**<.001**
Albumin, g/dL	3.50 (3.00, 4.00)	3.60 (3.10, 4.10)	2.90 (2.30, 3.40)	Z = −7.03	**<.001**
LAR	0.60 (0.40, 1.00)	0.50 (0.40, 0.90)	1.20 (0.80, 2.10)	Z = −8.74	**<.001**
LAR group, n(%)				χ² = 63.04	**<.001**
Low	528 (61.90)	510 (66.23)	18 (21.69)		
High	325 (38.10)	260 (33.77)	65 (78.31)		
**Comorbidities, n (%)**					
CHF				χ² = 14.31	**<.001**
No	672 (78.78)	620 (80.52)	52 (62.65)		
Yes	181 (21.22)	150 (19.48)	31 (37.35)		
Atrial fibrillation				χ² = 14.84	**<.001**
No	763 (89.45)	699 (90.78)	64 (77.11)		
Yes	90 (10.55)	71 (9.22)	19 (22.89)		
Respiratory failure				χ² = 94.76	**<.001**
No	654 (76.67)	626 (81.30)	28 (33.73)		
Yes	199 (23.33)	144 (18.70)	55 (66.27)		
Peripheral vascular				χ² = 0.47	0.495
No	787 (92.26)	712 (92.47)	75 (90.36)		
Yes	66 (7.74)	58 (7.53)	8 (9.64)		
Hypertension				χ² = 8.17	**0.004**
No	307 (35.99)	289 (37.53)	18 (21.69)		
Yes	546 (64.01)	481 (62.47)	65 (78.31)		
Renal disease				χ² = 3.84	0.050
No	594 (69.64)	544 (70.65)	50 (60.24)		
Yes	259 (30.36)	226 (29.35)	33 (39.76)		
Sepsis				χ² = 54.21	**<.001**
No	683 (80.07)	642 (83.38)	41 (49.40)		
Yes	170 (19.93)	128 (16.62)	42 (50.60)		
**Treatments n(%)**					
Vitamin B1				χ² = 6.34	**0.012**
No	798 (93.55)	715 (92.86)	83 (100.00)		
Yes	55 (6.45)	55 (7.14)	0 (0.00)		
Potassium Chloride				χ² = 2.16	0.141
No	128 (15.01)	111 (14.42)	17 (20.48)		
Yes	725 (84.99)	659 (85.58)	66 (79.52)		
Sodium Bicarbonate				χ² = 7.99	**0.005**
No	634 (74.33)	583 (75.71)	51 (61.45)		
Yes	219 (25.67)	187 (24.29)	32 (38.55)		
Vasopressin				χ² = 130.19	**<.001**
No	779 (91.32)	731 (94.94)	48 (57.83)		
Yes	74 (8.68)	39 (5.06)	35 (42.17)		
Mechanical ventilation				χ² = 1.71	0.191
No	841 (98.59)	761 (98.83)	80 (96.39)		
Yes	12 (1.41)	9 (1.17)	3 (3.61)		
**Disease severity score**					
SOFA	4.00 (2.00, 6.00)	3.00 (2.00, 5.00)	9.00 (5.00, 12.00)	Z = −10.26	**<.001**
APSIII	49.00 (38.00, 61.00)	47.00 (37.00, 59.00)	72.00 (56.50, 91.00)	Z = −9.32	**<.001**

SBP, systolic blood pressure; DBP, diastolic blood pressure; WBC, white blood cell; BUN, blood urea nitrogen; Scr, serum creatinine; LAR, lactate-to-albumin ratio; CHF, Congestive heart failure; SOFA,Sequential Organ Failure Assessment; APSIII, Acute Physiology Score III.

### 3.2 The connection between LAR and 28-day mortality risk

Covariates demonstrating remarkable differences (P < 0.05) presented in Table 1 were included in the single-factor Cox proportional hazards regression analysis, with the results shown in Table 2. Then, variables having P-values <0.05 in Table 2 were incorporated into the multivariate Cox regression model. Table 3 presents the outcomes of the Cox proportional hazards model analysis examining the connection between the LAR and all-cause in-hospital mortality within 28 days among DKA patients. The findings show that LAR acts as a risk factor that stands alone in the unadjusted model (P < 0.001), the partially adjusted model (P < 0.001), and the fully adjusted model (P < 0.001).

**Table 2:** Univariate cox regression analysis for 28-day mortality.

Variables	β	S.E	Z	*P*	HR (95%CI)
Age	0.05	0.01	6.68	**<.001**	1.05 (1.03 ~ 1.06)
Race					
White					1.00 (Reference)
Black	−0.33	0.30	−1.11	0.266	0.72 (0.40 ~ 1.29)
Other	0.85	0.25	3.37	**<.001**	2.33 (1.43 ~ 3.82)
SBP	−0.04	0.01	−5.64	**<.001**	0.96 (0.94 ~ 0.97)
DBP	−0.06	0.01	−5.39	**<.001**	0.95 (0.93 ~ 0.97)
Respiratory Rate	0.15	0.02	7.10	**<.001**	1.16 (1.11 ~ 1.20)
Vitamin B1					
No					1.00 (Reference)
Yes	−17.11	2118.30	−0.01	0.994	0.00 (0.00 ~ Inf)
Sodium Bicarbonate					
No					1.00 (Reference)
Yes	0.65	0.23	2.89	**0.004**	1.92 (1.23 ~ 2.98)
Vasopressin					
No					1.00 (Reference)
Yes	2.35	0.22	10.53	**<.001**	10.49 (6.77 ~ 16.24)
CHF					
No					1.00 (Reference)
Yes	0.84	0.23	3.70	**<.001**	2.32 (1.49 ~ 3.62)
Atrial fibrillation					
No					1.00 (Reference)
Yes	1.01	0.26	3.85	**<.001**	2.73 (1.64 ~ 4.56)
Respiratory failure					
No					1.00 (Reference)
Yes	2.00	0.23	8.61	**<.001**	7.39 (4.69 ~ 11.65)
Hypertension					
No					1.00 (Reference)
Yes	0.73	0.27	2.76	**0.006**	2.08 (1.24 ~ 3.51)
Sepsis					
No					1.00 (Reference)
Yes	1.51	0.22	6.88	**<.001**	4.53 (2.94 ~ 6.97)
Glucose	0.00	0.00	1.84	0.066	1.00 (1.00 ~ 1.00)
SOFA	0.26	0.02	12.23	**<.001**	1.30 (1.24 ~ 1.35)
APSIII	0.04	0.00	11.75	**<.001**	1.04 (1.03 ~ 1.04)
Hemoglobin	−0.17	0.05	−3.32	**<.001**	0.85 (0.77 ~ 0.93)
Platelets	−0.01	0.00	−4.54	**<.001**	0.99 (0.99 ~ 0.99)
Hematocrit	−0.04	0.02	−2.36	**0.018**	0.96 (0.93 ~ 0.99)
BUN	0.02	0.00	6.68	**<.001**	1.02 (1.02 ~ 1.03)
Scr	0.10	0.04	2.60	**0.009**	1.10 (1.02 ~ 1.19)
Sodium	0.07	0.01	5.11	**<.001**	1.07 (1.04 ~ 1.10)
Potassium	0.58	0.20	2.92	**0.003**	1.79 (1.21 ~ 2.66)
Chloride	0.05	0.01	4.24	**<.001**	1.05 (1.03 ~ 1.08)
LAR	0.28	0.03	10.12	<.001	1.32 (1.25 ~ 1.39)

HR: Hazard Ratio, CI: Confidence Interval.

SBP, systolic blood pressure; DBP, diastolic blood pressure; BUN, blood urea nitrogen; Scr, serum creatinine; LAR, lactate-to-albumin ratio; CHF, Congestive heart failure; SOFA,Sequential Organ Failure Assessment; APSIII, Acute Physiology Score III

**Table 3:** Multivariate Cox analysis of risk factors for 28-day mortality.

Variables	Multivariate Cox		
	HR	95%CI	*P*-value
**Model I**			
LAR	1.32	1.25 ~ 1.39	**<.001**
**Model II**			
LAR	1.28	1.17 ~ 1.40	**<.001**
Age	1.04	1.02 ~ 1.05	<.001
Race(Black)	0.92	0.51 ~ 1.67	0.789
Race(Other)	1.82	1.08 ~ 3.08	**0.024**
DBP	1.00	0.98 ~ 1.03	0.914
SBP	0.97	0.96 ~ 0.99	**0.002**
Respiratory Rate	1.08	1.03 ~ 1.14	**0.003**
CHF	0.92	0.56 ~ 1.51	0.736
Atrial fibrillation	1.14	0.64 ~ 2.03	0.647
Respiratory failure	2.66	1.60 ~ 4.43	**<.001**
Hypertension	1.13	0.63 ~ 2.01	0.686
Sepsis	1.56	0.98 ~ 2.47	0.061
**Model III**			
LAR	1.25	1.12 ~ 1.40	**<.001**
Age	1.04	1.02 ~ 1.06	**<.001**
Race(Black)	0.62	0.33 ~ 1.18	0.146
Race(Other)	1.44	0.82 ~ 2.51	0.201
DBP	1.02	0.99 ~ 1.04	0.242
SBP	0.98	0.96 ~ 0.99	**0.034**
Respiratory Rate	1.06	1.01 ~ 1.12	**0.020**
CHF	1.08	0.65 ~ 1.80	0.762
Atrial fibrillation	0.83	0.45 ~ 1.52	0.550
Respiratory failure	1.68	0.94 ~ 2.98	0.078
Hypertension	1.09	0.60 ~ 1.97	0.775
Sepsis	1.22	0.73 ~ 2.03	0.443
Hematocrit	1.10	0.94 ~ 1.29	0.228
Hemoglobin	0.75	0.47 ~ 1.19	0.216
Platelets	1.00	1.00 ~ 1.00	0.950
BUN	1.01	1.00 ~ 1.02	0.131
Scr	0.95	0.80 ~ 1.13	0.592
Sodium	1.00	0.95 ~ 1.06	0.888
Potassium	1.61	1.09 ~ 2.38	**0.016**
Chloride	1.02	0.98 ~ 1.06	0.442
Sodium Bicarbonate	0.48	0.27 ~ 0.86	**0.013**
Vasopressin	1.10	0.55 ~ 2.20	0.784
SOFA	1.10	0.99 ~ 1.23	0.067
APSIII	1.01	1.01 ~ 1.03	**0.035**

HR: Hazard Ratio, CI: Confidence Interval.

SBP, systolic blood pressure; DBP, diastolic blood pressure; BUN, blood urea nitrogen; Scr, serum creatinine; LAR, lactate-to-albumin ratio; CHF, Congestive heart failure; SOFA, Sequential Organ Failure Assessment; APSIII, Acute Physiology Score III

### 3.3 Analysis of ROC and K-M curve

**Fig 2:**
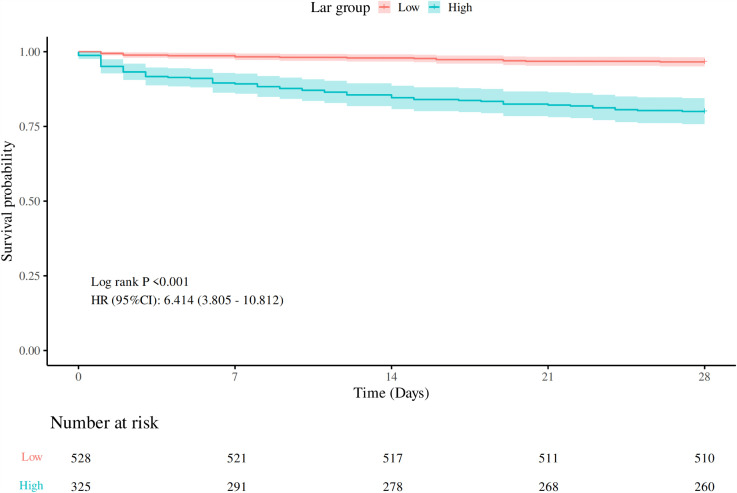
Kaplan-Meier survival curves for 28-day mortality among low LAR group and high LAR group.

Receiver operating characteristic（ROC） curves for the LAR, lactate, and albumin were constructed to predict 28-day mortality from all causes following admission in DKA patients. S1 Fig illustrates the ROC curves of LAR, lactate, and albumin. S1 Table in S1 File provides the AUC and 95% CI, cut-off values, sensitivity, and specificity of the three indicators. It can be observed that LAR possesses a remarkable predictive superiority, with the AUC of 0.79, which is evidently more advantageous than 0.75 of lactate and 0.73 of albumin. Meanwhile, the best threshold value for the LAR was found to be 0.75. Based on this best threshold value, DKA patients were stratified into a high-level LAR group (LAR ≥ 0.75, n = 325) and a low-level LAR group (LAR < 0.75, n = 528). The Kaplan-Meier method for survival analysis curve was drawn (Fig 2), demonstrating that the mortality rate in the high-LAR group was markedly elevated compared with the low-LAR group (P < 0.001).

### 3.4 The nonlinear relationship between LAR and 28-day all-cause mortality in DKA patients (Fig 3)

**Fig 3:**
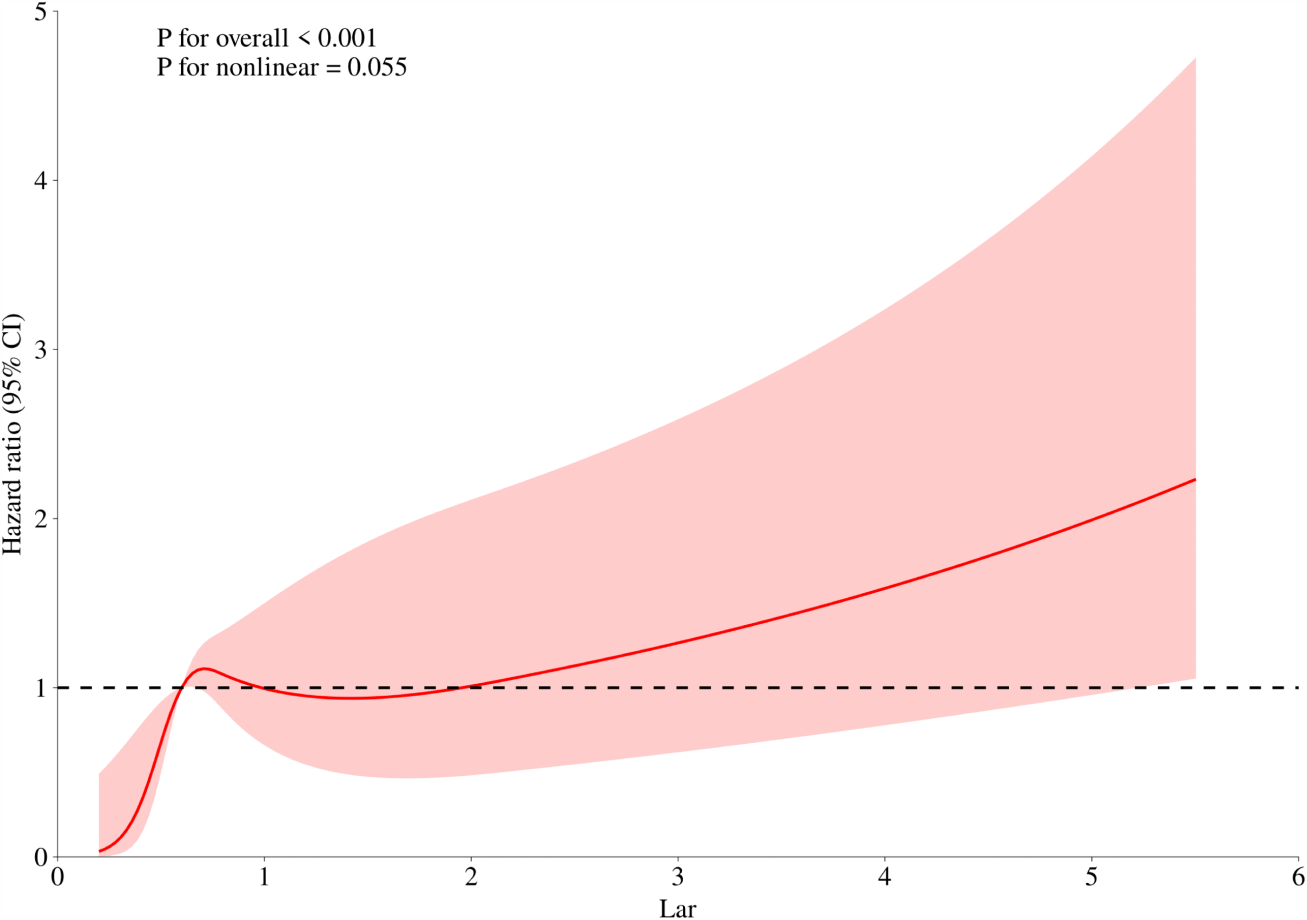
RCS curve for the LAR hazard ratio and 28-day mortality.

The model of RCS revealed a non-linear association among LAR and the 28-day mortality caused by all reasons in DKA patients (Fig 3). The research results indicated that when LAR remained as a continuous variable, the 28-day mortality rate at admission might have a linear association with LAR, increasing linearly with the increase of LAR (non-linear P = 0.055).

### 3.5 Subgroup analysis (Fig 4)

**Fig 4:**
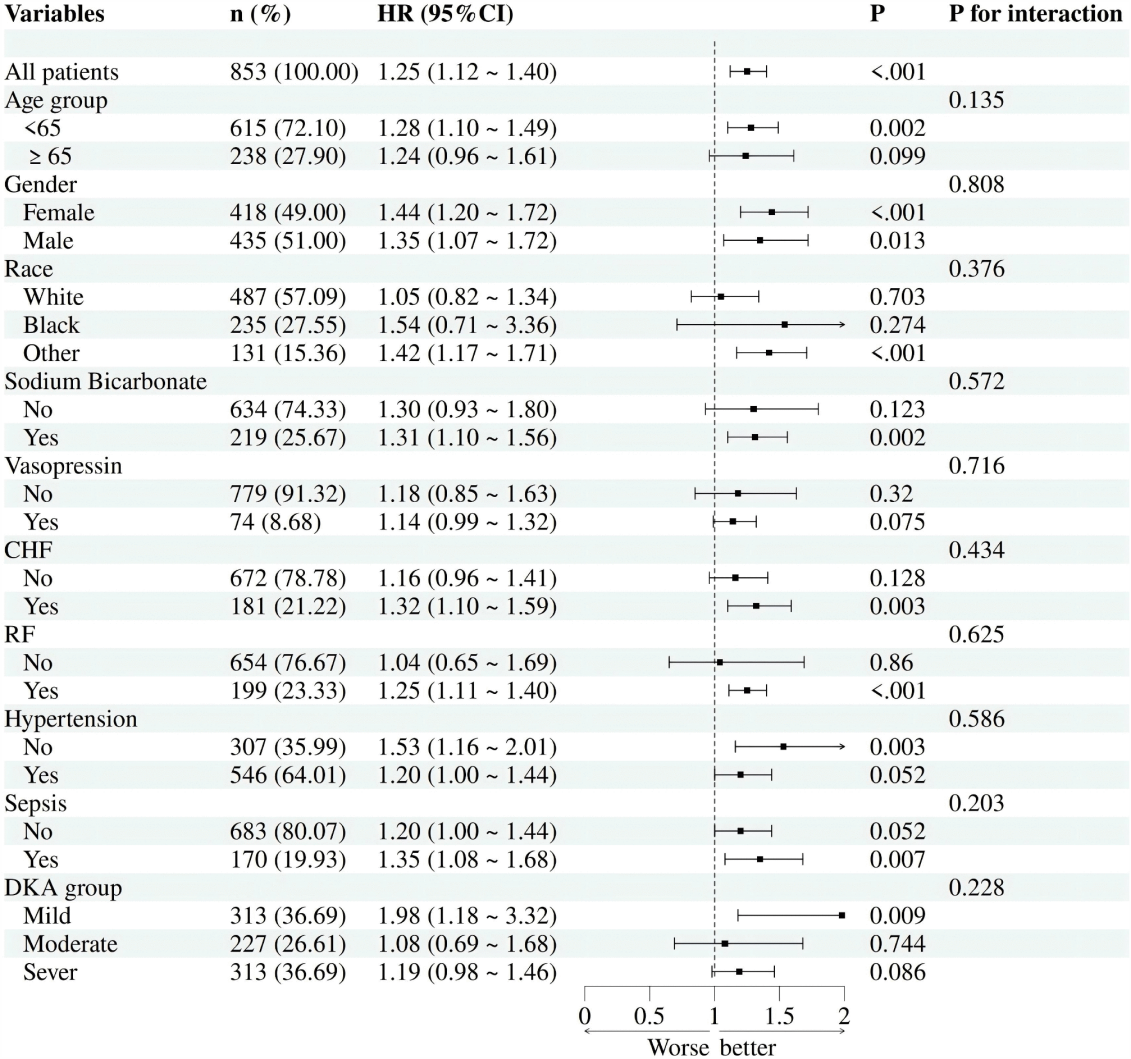
Forest plots for different subgroup analysis of HR for the association between LAR and 28-day mortality.

To explore whether the result is stable across different populations, we stratified the study by age, gender, race, sodium bicarbonate, vasopressin, congestive heart failure, respiratory failure, hypertension, and sepsis. Consistent results were observed in all subgroups (Fig 4), that is, the interaction test between covariates and LAR did not show significance(P > 0.05), which proved that LAR was a prognostic factor that was independent.

## 4 Discussion

Our research indicates that the 28-day all-cause mortality of DKA patients in the high LAR group markedly increased (P < 0.05). Similar to the CAR in sepsis-associated AKI [12], our findings demonstrate that LAR integrates metabolic (lactate) and inflammatory (albumin) derangements to improve risk prediction. However, unlike CAR’s J-shaped association with mortality, LAR exhibited a linear relationship (non-linear P = 0.055), suggesting distinct pathophysiological mechanisms. Following adjustment for potential confounders, Cox regression analysis revealed that an increased LAR (≥ 0.75) independently predicted an elevated risk of 28-day all-cause mortality in DKA patients (P < 0.001). The K-M survival analysis revealed that patients of the high LAR group had a markedly reduced 28-day cumulative survival rate compared to patients in the low LAR group (P < 0.001). Subgroup analyses further validated the robustness of the study findings. In the subgroup analysis, based on the grading criteria for bicarbonate concentration as reported in the 2024 Consensus Report on the Adult Diabetic Hyperglycemia Crisis [13], we further divided the DKA patients into three subgroups: mild, moderate, and severe, and explored the predictive value of LAR in these different severity subgroups. The results showed that there was no significant interaction between LAR and the severity of DKA (interaction P = 0.228), suggesting that the predictive effect of LAR was relatively consistent across different subgroups. It is worth noting that LAR demonstrated significant predictive value in the mild DKA subgroup (P = 0.009), but did not reach statistical significance in the moderate (P = 0.744) and severe (P = 0.086) subgroups. We believe that patients with moderate to severe DKA often have more severe acidosis, dehydration, and inflammation than those with mild DKA, and these acute factors may partially mask the true association of LAR with prognosis. Second, heterogeneity in clinical characteristics across severity subgroups may also have had an impact on the results. Although we adjusted for major confounders, residual confounding is inevitable. This finding suggests that LAR shows good predictive ability in patients with mild DKA, while its value in moderate-to-severe patients needs to be further verified by studies with larger sample sizes. In the future, the severity of DKA may need to be considered when applying LAR for risk stratification in order to achieve more accurate prognosis assessment. In addition, it should be noted that confidence intervals were wide for some subgroups (e.g., patients with black race, no vasopressin use, and no concomitant respiratory failure). This limitation is mainly related to the small sample size in the corresponding subgroups: Black race and respiratory failure were underrepresented in the total cohort, and vasopressin was used in relatively few cases because of its strict clinical indications, which resulted in underpowered statistical tests. Future studies should expand the sample size or design specific studies for the above population to further improve the reliability and power of subgroup analysis. Furthermore, the ROC curve revealed that the LAR exhibited significant diagnostic performance in predicting the prognosis of patients with DKA (AUC = 0.79). In addition, we used the Youden index to determine the optimal cut-off value of LAR for predicting the risk of mortality in DKA patients was 0.75, patients with LAR above this value had a significantly increased risk of poor prognosis, which was consistent with the findings of Liu et al. in patients with acute pancreatitis (LAR cutoff value was 1.1124) [14]. Although the research subjects are different, both of them involve similar pathophysiological processes such as metabolic disorders and systemic inflammatory response. This cross-disease corroboration further supports the validity of LAR as a prognostic indicator for DKA, and also reflects the reliability of the cut-off value determined in this study. Therefore, LAR can be used as a simple and feasible indicator to recognize high-risk DKA patients in the early stage and take active intervention measures to improve the prognosis of patients.

Diabetic ketoacidosis as an acute diabetic complication, has attracted significant attention in the last few years due to the increasing global prevalence of diabetes, emerging as a substantial challenge in healthcare systems worldwide. Numerous researchers have embarked on endeavors to investigate the significance of diverse biomarkers in forecasting the prognoses for patients with DKA. The study results demonstrate that red blood cell distribution width (RDW) and the ratio of RDW to albumin (RDW/ALB), and the ratio of serum urea nitrogen to serum albumin (BUN/ALB) are crucial biomarkers to forecast the outcome of DKA patients [14–16]. High lactate levels have been demonstrated to be related to unfavorable prognosis in DKA patients [17]. Although lactate indicators can reflect tissue hypoxia and metabolic disorders, its production is not only due to tissue hypoxia, but abnormal liver metabolism can also lead to an increase in its level [18,19]; In addition, the use of biguanide drugs and β2 receptor agonists, such as epinephrine, can also promote glycolysis, thereby increasing the production of lactate [20,21]. Therefore, relying merely on lactate as the only predictive indicator is unreliable. Decreased albumin levels serve as an indicator of the extent of inflammation and oxidative stress. Patients with DKA frequently exhibit a systemic inflammatory response and a state of physiological stress, potentially leading to a reduction in albumin levels. However, albumin concentrations are susceptible to various of factors, including malnutrition [22,23], and using albumin alone for prognosis assessment has certain limitations. LAR combines the pathophysiological significance of lactate and albumin, enabling a more holistic appraisal of the pathophysiological state of DKA patients and providing more accurate predictions than using the lactate or the albumin independently [24]. Furthermore, lactate as well as albumin are routinely measured parameters, and the calculation of the LAR is straightforward and easily implementable, rendering it highly suitable for broad clinical use.

The precise mechanisms by which the LAR influences the predicted outcome of patients in critical condition with DKA remain unclear. Existing research holds that this is related to tissue hypoxia, hyperglycemic status and secondary inflammation in DKA patients. Lactate is the end product of glycolysis. In an oxygen-deficient environment, pyruvate cannot be further transformed in the citric acid cycle, and must be reduced to lactate by lactate dehydrogenase with the help of nicotinamide adenine dinucleotide (NADH) [25]. In DKA, insulin deficiency, impaired glucose utilization, and insufficient cellular energy supply occur, leading to a reliance on anaerobic glycolysis for lactate production. The osmotic diuresis induced by hyperglycemia will accelerate the loss of water, various minerals and electrolytes, causing a reduction in blood volume and poor tissue perfusion, and intensifying tissue hypoxia [26]. Furthermore, due to insulin deficiency, the elevation in counter-regulatory hormones, particularly epinephrine, which promotes the activity of hormone-sensitive lipase within adipose tissue, resulting in enhanced fat breakdown such as triglycerides in DKA [27]. A large amount of NADH is produced during the process of fatty acid metabolism. The high concentration of NADH further promotes the generation of lactate [25]. Approximately 70% to 75% of lactate undergoes hepatic metabolism [28], owing to the influences of acidosis and dehydration, the liver function of patients with DKA might be temporarily compromised, thereby lowering the clearance rate of lactate. Research has shown that elevated lactate levels could be observed in liver diseases [19,29]. Hypoalbuminemia acts as a marker of the degree of the extent of systemic inflammation. Clinical research has shown that the progression of hyperglycemia and DKA is related to the rise in levels of pro-inflammatory cytokines and the growth of oxidative stress markers [30,31], thereby leading to enhanced capillary permeability [32]. Albumin kinetics encompasses leakage and rupture across capillaries, resulting in hypoalbuminemia [22]. Furthermore, patients with DKA are frequently accompanied by anorexia and liver dysfunction. Since albumin is predominantly produced by the liver [33], the substantial reduction in both albumin intake and synthesis contributes to the decline in albumin levels in DKA patients. Besides, the elevation of lactate is able to take part in the course of inflammatory damage within the body and facilitate the inflammatory reaction [34]. Meanwhile, albumin has a certain function in anti-inflammation and maintaining acid-base equilibrium. It can mitigate tissue damage induced by inflammatory processes and promote wound healing through the enhanced production of anti-inflammatory mediators, including resolvins, lipoxins, and protectins [35]. Evidently, a high LAR indicates cellular hypoxia, inadequate blood perfusion, metabolic disorders of the organism, and severe inflammatory reactions, a higher concentration of lactate and a lower level of albumin can intensify the damage to the organism caused by inflammation, thereby resulting in a higher mortality rate among DKA patients. Thus, the selection of the LAR as a predictor for DKA is both feasible and clinically relevant. In our research, accuracy analyses were performed on lactate, albumin, and LAR respectively. It was discovered that the AUC of LAR was 0.79, which was evidently superior to the individual application of lactate and albumin. It can thus be observed that the ratio of lactate to albumin varies inversely through two distinct mechanisms, which can significantly diminish the impact of individual factors on the regulatory mechanism, thereby facilitating a more accurate prediction of the prognosis in DKA patients.

The American Journal of Emergency Medicine put forward in 2020 that LAR could act as an initial prognostic indicator for severely patients in the ICU, and it was verified that the LAR at admission could forecast severe sepsis or septic shock independently [36].Subsequently, numerous studies encompassing different disease domains have indicated that LAR can be utilized as a predictive indicator for disease mortality. Wang et al. discovered that despite the fact that the predictive capability of LAR for the outcome of patients having liver cirrhosis in association with sepsis was a bit less than that of the Model for End-Stage Liver Disease score, it was conspicuously superior to those of albumin, lactate, and SOFA [37]. Liu et al. indicated that LAR might function as a separate prognostic indicator in terms of the 28-day all-cause mortality in individuals with acute pancreatitis following hospital admission [9]. One study has disclosed the predictive effect of LAR on acute respiratory distress syndrome, verifying the existence of a positive correlation between LAR and 28-day mortality [38]. Furthermore, it has been verified that LAR is remarkably connected to the prognosis of traumatic brain injury and acute kidney injury [39,40]. The aforementioned studies have verified that the predictive capacity of LAR exceeds the predictive effect of using lactate or albumin separately, which is in line with our research discoveries. Evidently, with respect to the prognosis of patients with severe diabetic ketoacidosis, attaching significant importance to the LAR has significant clinical implications.

Based on the results of this study, we recommend the use of LAR as an important biomarker for early risk assessment in DKA patients with a stratified management strategy. Medical institutions can incorporate LAR testing into the admission evaluation system for DKA patients, integrate LAR automatic calculation capabilities into the electronic medical record system, and establish early warning mechanisms. For high-risk patients with LAR ≥ 0.75, an ICU consultation alert was automatically triggered and they were transferred to intensive care if necessary. At the same time, it is recommended to implement the dynamic monitoring program of lactate and albumin for high-risk patients, evaluate the treatment effect and disease evolution in time, and dynamically adjust the treatment strategy. For patients with significantly elevated LAR (≥0.75), tissue hypoperfusion should be corrected first, and adequate fluid resuscitation should be completed before starting insulin therapy [41]. For patients with persistent metabolic acidosis (arterial pH < 7.1) and persistently elevated LAR, it is recommended to consult nephrologists as soon as possible to evaluate the indications for renal replacement therapy [42]. For patients who are at risk for elevated LAR but whose initial values do not meet high-risk criteria (<0.75), preventive interventions should be implemented. For patients with signs of infection, it is recommended to complete etiological examination as soon as possible after admission and select sensitive antibiotics according to drug sensitivity results to prevent further increase of lactate. For patients with malnutrition or chronic liver disease, serum albumin levels should be dynamically monitored, and intravenous supplementation of human serum albumin should be considered when serum albumin is less than 30 g/L to prevent further decrease in albumin [43]. Patients were given health education intervention and taught to identify the deterioration signs such as persistent nausea, deepening abdominal pain, and respiratory rate >25 times/min [44]. Establish a standardized blood glucose monitoring system, standardize the insulin treatment plan, especially emphasize that the dose should not be adjusted by oneself under stress. The implementation of smoking and drinking cessation in lifestyle, and the diet was reasonable and serum albumin levels were measured every 3–6 months. The above strategies can effectively improve the long-term prognosis and quality of life of patients, and reduce the incidence of ketoacidosis events and adverse outcomes.

## 5 Limitations

In this study, multiple advanced statistical approaches such as multivariate Cox regression analysis, ROC curve analysis, survival analysis, RCS analysis along with subgroup analysis were utilized to comprehensively evaluate the predictive potency that LAR has for the prognosis of DKA patients. Nevertheless, this research has its own limitations. First, as a retrospective research carried out in a single center, the potential for selective bias remains a possibility that cannot be dismissed. The generalizability of the findings derived from this database to broader populations cannot be assured. Secondly, we conducted the analysis solely on the basis of the LAR value measured at the beginning stage of admission and didn’t deeply explore the influence of the dynamic changes of LAR on the outcome of DKA patients. Meanwhile, the levels of lactate as well as those of albumin are influenced by multiple factors, and we cannot completely exclude the possible interference of additional factors to these indicators. For example, because the MIMIC-IV database does not systematically document preadmission alcohol use, we were unable to identify and exclude patients who had recently consumed alcohol before admission, which could have had an effect on lactate levels. In addition, several emerging composite indicators, such as the triglyceride-glucose index (TyG) [45], and neutrophil-to-PNI ratio [46], have demonstrated promising predictive efficacy in the prognosis of sepsis. Future research is warranted to compare the predictive performance of LAR with these indicators. Therefore, future research should be conducted as multi-center studies, prospective studies to further validate the predictive significance of LAR and deeply investigate the relationship between its dynamic changes and prognosis.

## 6 Conclusions

The findings of this research reveal that LAR acts as an individual predictor of the 28-day prognosis for DKA patients upon admission, and its predictive performance exceeds that of lactate or albumin levels alone. LAR offers a practical and efficient method, facilitating the early detection of high-risk diabetic ketoacidosis patients and supporting clinicians in tailoring individualized treatment strategies.

## Supporting information

S1 Fig The ROC curves of LAR, lactate, and albumin. (TIF)

S1 File The AUC and 95% CI, cut-off values, sensitivity, and specificity of the three indicators. (PDF)

S2 File Original data. (CSV)

S3 File Calculation of coefficient of variation. (CSV)
