# Supplementary figures and images for: Association between the lactate-to-albumin ratio and 28-day all-cause mortality in diabetic ketoacidosis patients: A retrospective cohort study utilizing the MIMIC-IV database
Source: PLoS One. 2026 Mar 12;21(3):e0344767. doi: 10.1371/journal.pone.0344767 (PMC12981510; doi:10.1371/journal.pone.0344767)

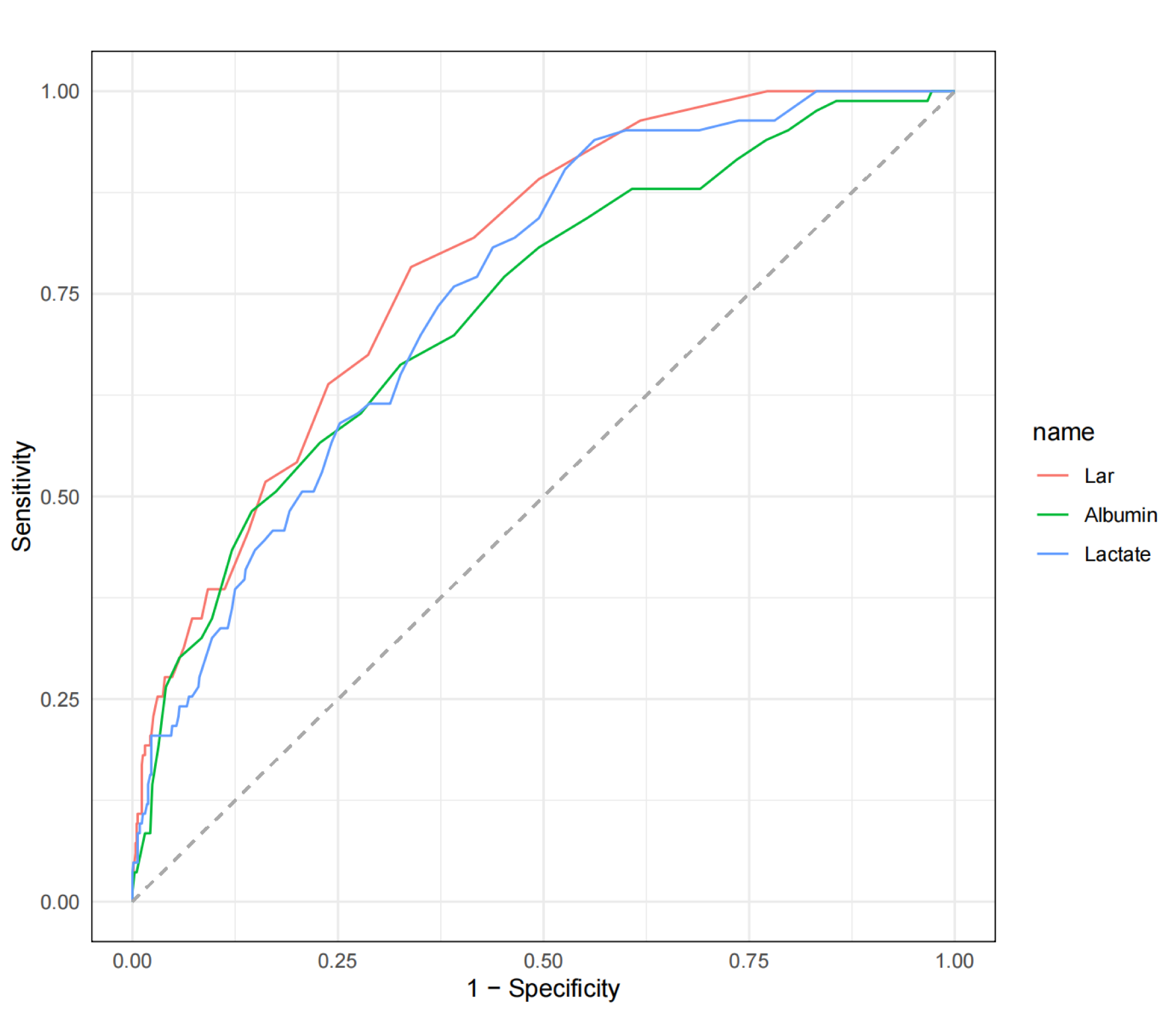

Supplement: S1 Fig — (TIF) [file pone.0344767.s001.tif]
