# Supplementary material for: Association between the lactate-to-albumin ratio and 28-day all-cause mortality in diabetic ketoacidosis patients: A retrospective cohort study utilizing the MIMIC-IV database
Source: PLoS One. 2026 Mar 12;21(3):e0344767. doi: 10.1371/journal.pone.0344767 (PMC12981510; doi:10.1371/journal.pone.0344767)
Supplement: S1 File — (PDF) [file pone.0344767.s002.pdf]

Variables	AUC	95%CI	Threshold	Sensitivity	Specificity
LAR	0.79	0.74-0.84	0.75	0.66	0.78
Lactate	0.75	0.70-0.80	1.75	0.44	0.94
Albumin	0.73	0.68-0.79	3.05	0.77	0.57
